# Are the Global Strategic Directions for Strengthening Nursing and Midwifery 2016–2020 being implemented in countries? Findings from a cross-sectional analysis

**DOI:** 10.1186/s12960-019-0392-2

**Published:** 2019-07-12

**Authors:** Onyema Ajuebor, Carey McCarthy, Yin Li, Sumaya Mohamed Al-Blooshi, Nonhlanhla Makhanya, Giorgio Cometto

**Affiliations:** 1 Health Workforce Department, World Health Organization, 20 Avenue Appia, CH-1211 Geneva 27, Switzerland; 2 Nell Hodgson Woodruff School of Nursing, Emory University, Atlanta, USA; 3 Nursing Department, Ministry of Health and Prevention, Abu Dhabi, UAE; 4 National Department of Health, Pretoria, South Africa

**Keywords:** Nurses, Midwives, Health policy, Implementation, Health systems

## Abstract

**Background:**

Nurses and midwives are the largest component of the health workforce in many countries. The World Health Organization (WHO) together with its partners facilitates the joint development of strategic policy guidance for countries to support the optimization of their nursing and midwifery workforce. The Global Strategic Directions for Strengthening Nursing and Midwifery 2016–2020 (SDNM) is a global policy guidance tool that provides a framework for Member States, the WHO and its partners to adapt, develop, implement and evaluate nursing and midwifery policy interventions in Member States. As part of the broader monitoring and accountability functions of the WHO, assessing the progress of the SDNM implementation at a country level is key to ensuring that countries stay on track towards achieving universal health coverage (UHC) and the sustainable development goals (SDGs).

**Methods:**

This is a cross-sectional mixed methods study involving the analysis of quantitative and qualitative data on the implementation of country-level interventions in the SDNM. Data was provided by government chief nursing and midwifery officers or their representatives using an online self-reported questionnaire. The quantitative data was assessed using a three-level scale and descriptive statistics while qualitative comments were analysed and presented narratively.

**Results:**

Thirty-five countries completed the survey. Summing up the implementation frequency of interventions across all thematic areas, 19% of responses were in the category of “completed”; 55% were reportedly “in progress” and 26% indicated a status of “not started”. Findings reveal a stronger level of implementation for areas of nursing and midwifery development related to enhancing regulation and education, creating stronger roles for professional associations and policy strengthening. Leadership and interprofessional collaboration are intervention areas that were less implemented.

**Conclusion:**

Monitoring and accountability of countries’ commitments towards implementing nursing and midwifery interventions, as outlined in the SDNM, contributes to strengthening the evidence base for policy reforms in countries. This stock-taking can inform policy- and decision-makers’ deliberations on strengthening the contributions of nurses and midwives to achieving UHC and the SDGs.

**Electronic supplementary material:**

The online version of this article (10.1186/s12960-019-0392-2) contains supplementary material, which is available to authorized users.

## Background

The nursing and midwifery workforce comprises approximately half of the global health workforce and optimizing their role can contribute to the progressive realization of universal health coverage (UHC) and the Sustainable Development Goals (SDGs) [1–3]. Despite the centrality of nurses and midwives and other allied health workers to advancing health and wellbeing [4–10], they have consistently experienced a myriad of challenges, such as shortages and maldistribution, low levels of retention and high levels of migration and ineffective policies and management [11–13]. Among these, some issues are particularly pronounced for the nursing and midwifery occupational groups. These include a lack of representation in policy leadership, health governance and decision-making positions [14], gender bias and low pay in the workplace [15] and regulatory frameworks that do not facilitate optimized scopes of practice nor lifelong learning. Education of the nursing and midwifery workforces is not always competency-based, interdisciplinary or supported by quality assurance mechanisms such as accreditation requirements and updated standards for educators and curricula [16]. The combination of challenges to the regulation, leadership, education and practice of nursing and midwifery has resulted in the undervaluation and often low social status of the professions [17].

The World Health Organization (WHO) leads collaboration with countries and the global health community on efforts to achieve SDG 3, including the target of UHC. UHC means that all individuals and communities have access to the health services they need without incurring financial hardship [18]. In 2018, the global community re-committed to primary health care (PHC) as the cornerstone of UHC. This centres around providing integrated people-centred health services that require interdisciplinary health workforce approaches and PHC policies and solutions that are equitable and responsive to communities’ needs [4, 5, 19]. Also, achieving UHC and the SDGs through strengthening health worker contribution is at the heart of the Global Strategy on Human Resources for Health: Workforce 2030 (GSHRH). The GSHRH provides a comprehensive framework for strengthening the health workforce across all disciplines and includes four overarching objectives related to (1) policies for health worker performance, education, accreditation and regulation, (2) concerted and strategic investments in the health workforce, (3) institutional capacity and governance and (4) health workforce data and monitoring [20]. WHO Member States adopted the GSHRH in 2016 inclusive of global milestone targets for 2020 and 2030 and an agreement to progressively implement National Health Workforce Accounts (NHWA) [21]. NHWA are country-owned health workforce information systems that support standardized, systematic and interoperable collection of relevant health workforce information to guide planning and policy development as well as track HRH policy performance towards achieving UHC [22].

Addressing the specific obstacles to optimized contributions of nurses and midwives to UHC and PHC goals was the intent behind the Global Strategic Directions for Strengthening Nursing and Midwifery 2016–2020 (SDNM) [23]. Developed through a series of consultative meetings in 2015 and 2016, the process involved over one hundred stakeholders from all WHO geographical regions. The SDNM were launched in 2016 at the Seventh Global Forum for Government Chief Nursing and Midwifery Officers (GCNMOs). The SDNM comprises four thematic areas (TA); each TA has a main objective and between four and seven specific interventions at the country-level to help achieve the objective of the TA. To understand the progress or challenges of Member States and accelerate implementation efforts in advancing the SDNM in their countries, GCNMOs attending the Eighth Global Forum for GCNMOs in 2018 were invited to share their progress in implementing the SDNM. A special session was held at the eighth forum to discuss the preliminary results. This paper presents a synthesis of the findings shared by GCNMOs, an analysis of the strengths and weaknesses in implementing the SDNM in the context of the SDGs, and it identifies opportunities for future policy reforms of the nursing and midwifery workforce.

## Methods

### Instrument

A 15-question, self-reported online questionnaire was developed and re-produced in three other languages (French, Russian and Arabic) (See Additional file 1). Its questions included respondents’ demographics (country, position and gender), whether or not they used the SDNM and if nursing and midwifery policy frameworks were used in their country. Respondents were asked to indicate the implementation status on the 22 interventions in the SDNM, categorized according to the four thematic areas (TAs), using a three-level scale (“not started”, “in progress” and “completed”). Open-ended questions asked about areas of need/support in implementing the SDNM. Respondents were also asked to share up to two case studies of how the SDNM were used to advance nursing and midwifery development in their countries.

### Procedure

The progress report questionnaire was distributed to the 78 GCNMOs or their representatives who confirmed attendance to the 2018 Forum. The GCNMOs received an email with a link to a Survey Monkey (subscription version) instrument. Participants had approximately 5 weeks to share their feedback—2 weeks before the meeting and 3 weeks after. Participation was voluntary and respondents were asked by email and through the survey tool for their consent to participate and for reported data to inform WHO governing bodies’ decisions and future nursing and midwifery policy guidance development.

### Analysis

Responses to the intervention questions were compulsory and only one answer was allowed per intervention, making a total of 770 possible answers overall. The answers were then divided into the appropriate categories (“not started”, “in progress” and “completed”) for each TA, generating frequency distributions for each intervention. Using Microsoft Excel 2010, the percentage value by category for each intervention was calculated and presented in a graphical format according to each TA. The responses to open-ended questions about needs, support areas, success areas and case study highlights were analysed and presented narratively.

## Results

Thirty-five responses were collected from the 78 attending participants giving a response rate of 45%. All six WHO regions were represented in the respondents; however, the greatest number of respondents were from the European (*n* = 11; 31%) and African (*n* = 10; 29%) regions, and only one respondent was from the Southeast Asian region (Table 1). Most respondents (*n* = 30; 86%) held the position of Chief Nursing or Chief Midwifery Officer; five respondents (14%) reported holding other positions, such as ministerial adviser (*n* = 1), coordinating (*n* = 1) or educational positions (*n* = 3). Regional frameworks on nursing and midwifery were reportedly available in 26 of the responding countries (57%) but implemented in only 14 (43%). Almost three quarters (*n* = 26; 74%) of respondents indicated using the SDNM to guide nursing and midwifery policy development in their setting. Of those reporting use of the SDNM, the clear majority (23; 88%) reported the SDNM were helpful.

**Table 1:** Description of respondent characteristics

Characteristic	*n* (%)
Position held	
Chief nurse/midwife	30 (86%)
Other position	5 (14%)
Gender	
Female	30 (86%)
Male	5 (14%)
WHO regional representation	
Africa	10 (29%)
Americas	5 (14%)
Eastern Mediterranean	2 (6%)
Europe	11 (31%)
Southeast Asia	1 (3%)
*Western Pacific	6 (17%)
Use of the SDNM 2016–2020	
Yes	26 (74%)
No	9 (26%)
**†**Usefulness of the SDNM 2016–2020	
Useful	23 (88%)
Uncertain	3 (12%)
Not useful	0 (0%)
Regional framework on nursing and midwifery	
Available and is implemented	15 (43%)
Available but not implemented	5 (14%)
Not available	15 (43%)

*Includes one special administrative region

†Includes only respondents who answered “yes” to having used the SDNM

We analysed together the 770 responses by 35 countries to the 22 country interventions to determine the average across all four thematic areas (TAs) (Table 2). Overall, 19% (*n* = 150) of responses were in the category of “completed”; 55% (*n* = 420) were reportedly “in progress” and 26% (*n* = 200) indicated a status of “not yet started”. TAs 1 and 2 were above the average in terms of the percent of responses categorized as “completed” or “either in progress and completed”. TAs 3 and 4 were below the average in these categories. The most progress in terms of percent of interventions reported as “completed” was in TA 1 at 25% and TA 2 with 22%. In TA 3, only 8% of interventions were reported as “completed”, and TA 3 had the highest relative percentage of interventions (*n =* 46; 33%) reported as “not started”. TA 4 also had a relatively high percent (30%) of interventions not started.

**Table 2:** Percent of interventions reported as “not started”, “in progress” and “completed” by thematic area

Thematic area	No. of interventions	No. of responders	Not started *N* (%)	In progress *N* (%)	Completed *N* (%)	Either in progress and completed
1. Ensuring an educated, competent and motivated nursing and midwifery workforce within effective and responsive health systems at all levels and settings	7	35	61 (25%)	123 (50%)	61 (25%)	184 (75%)
2. Optimizing policy development, effective leadership, management and governance	6	35	42 (20%)	122 (58%)	(46) 22%	168 (80%)
3. Working together to maximize the capacities and potentials of nurses and midwives through intra- and interprofessional collaborative partnerships, education and CPD	4	35	46 (33%)	77 (59%)	(10) 8%	87 (67%)
4: Mobilizing political will to invest in building effective evidence-based nursing and midwifery workforce development	5	35	53 (30%)	93 (53%)	(30) 17%	123 (70%)
Overall from four thematic areas	22	35	200 (26%)	420 (55%)	(150) 19%	570 (74%)

### Findings by thematic area

#### Thematic area 1: Ensuring an educated, competent and motivated nursing and midwifery workforce within effective and responsive health systems at all levels and in different settings

**Fig. 1:**
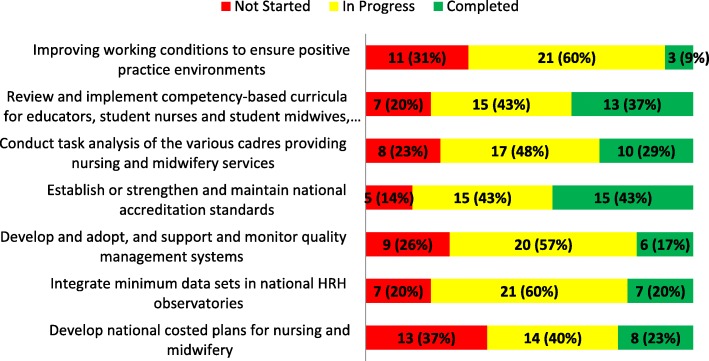
Progress reported on interventions in thematic area 1. The figure shows the progress made in the implementation of country-level interventions in thematic area one of the SDNM 2016–2020

Within this TA, the interventions with the highest number of countries reporting as “completed” were, *Establishing or strengthening and maintaining national accreditation standards* (*n =* 15; 43%) and *Reviewing and implementing competency-based curricula* (*n =* 13; 37%) (Fig. 1). In addition, these two interventions were the first and third, respectively, most “completed” interventions across all four TAs. When responses of “completed” were combined with the responses of “in progress”, 86% of responding countries indicated they had at least begun the work around accreditation and 80% have begun the work on competency-based curricula. Similarly, 80% of responding countries also indicated that they had either completed (*n =* 7; 20%) or were “in progress” (*n =* 21; 60%) on *Integrating minimum data sets (MDS) into national HRH observatories or information systems.* The intervention reported to be the most “not started” was *Developing national costed plans for nursing and midwifery development* (13; 37%), followed by, “*Improving working conditions to ensure positive practice environments*” (11; 31%).

#### Thematic area 2: Optimizing policy development, effective leadership, management and governance

**Fig. 2:**
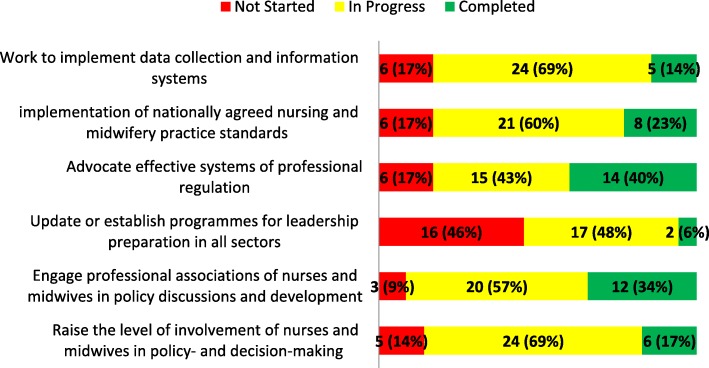
Progress reported on interventions in thematic area 2. The figure shows the progress made in the implementation of country-level interventions in thematic area two of the SDNM 2016–2020

The responses to the status of interventions in TA 2 were also analysed separately (Fig. 2). The intervention that was most commonly reported as “completed” in TA 2 was, *Advocating for effective regulations and the legislative authority to implement them* (*n =* 14; 40%). This was ranked the second highest “completed” intervention across all four TAs. The intervention with the highest number of “completed” and “in progress” status combined was, *Engaging professional associations in policy discussions and development* (*n =* 32; 91%); this was tied for the highest percentage across all four TAs. Similar to the findings about developing a minimum data sets (MDS) for nursing and midwifery workforce data in TA 1, an almost corresponding number (*n =* 29; 83%) reported *Implementing data collection and information systems* in TA 2 as either “completed” or “in progress”. While the intervention, *Raising the level of nurses and midwives in policy- and decision-making*, had a high percentage of countries reporting it as “in progress” or “completed” (*n =* 30; 86%), the intervention, *Update or establish programmes for leadership preparation*, had a relatively low number of countries reporting this as “in progress” (*n =* 17; 48%) and the lowest number reporting “completed” (*n =* 2; 6%) in TA 2.

#### Thematic area 3: Working together to maximize the capacities and potentials of nurses and midwives through intra- and interprofessional collaborative partnerships, education and continuing professional development

**Fig. 3:**
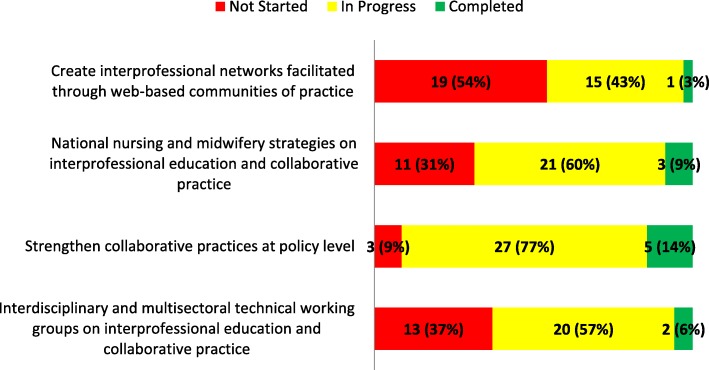
Progress reported on interventions in thematic area 3. The figure shows the progress made in the implementation of country-level interventions in thematic area three of the SDNM 2016–2020

The responses by countries on the interventions in TA 3 were analysed according to their individual category status and by combining the “in progress” and “completed” responses (Fig. 3). The intervention that most countries (*n =* 19; 54%) reported as “not started” was *Create interprofessional networks facilitated through web-based communities of practice*; this intervention had the fewest (*n =* 1; 3%) countries reporting it as “completed” across all four TAs. On the contrary, TA 3 also had the highest number overall (*n =* 32; 91%) reporting “in progress” and “completed” for the intervention, *Strengthening collaborative practices at policy level*. This intervention was reported as “in progress” (*n =* 27; 77%), the highest across all four TAs.

#### Thematic area 4: Mobilizing political will to invest in building effective evidence-based nursing and midwifery workforce development

**Fig. 4:**
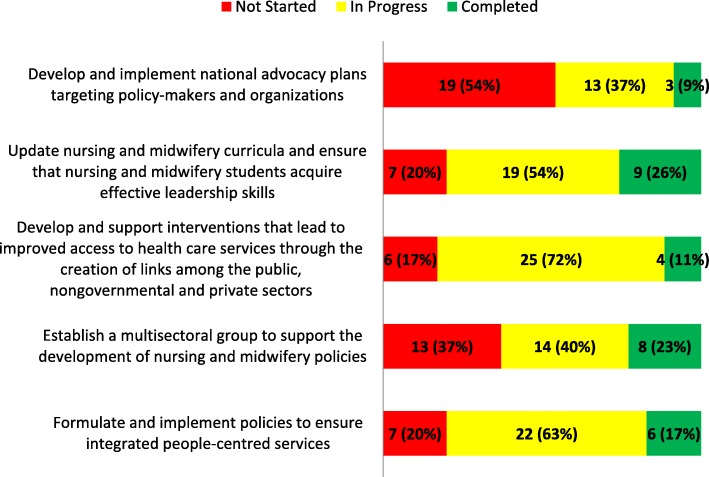
Progress reported on interventions in thematic area 4. The figure shows the progress made in the implementation of country-level interventions in thematic area four of the SDNM 2016–2020

Responses describing the status of interventions in TA 4 were analysed similarly to the other TAs (Fig. 4). The intervention reported most as “completed” in this TA was, *Update nursing and midwifery curricula so students acquire leadership skills and the ability to influence policy* (*n =* 9; 26%). The intervention with the most responses reflecting “in progress” and “completed” combined was *Improving access to health care services through linking the public, NGO and private sectors* (*n =* 29; 83%). When “in progress” and “completed” interventions are combined, 80% (*n =* 28) of countries reported the intervention, *Ensure integrated people-centred health services*. The intervention, *Develop and implement national advocacy plans to target policy-makers and organizations*, tied with the TA 3 intervention, *Create interprofessional networks facilitated through web-based communities of practice*, for the highest number of interventions across all TAs of “not started” (*n =* 19; 54%).

## Discussion

We collected self-reported information from 35 countries attending the eighth GCNMO Forum on the status (“not started”, “in progress”, “completed”) of 22 country-level interventions included in the SDNM. The top three interventions marked as “completed” were, *Advocating for effective regulations and the legislative authority to implement them*, *Establishing or strengthening national accreditation standards* and *Reviewing and implementing competency-based curricula*. The reported progress on regulation and accreditation is a positive indication of the role of regulatory bodies in ensuring that health workers in the public and private sectors of countries are competent and meet established standards needed to practice. Effective regulations promoting access to comprehensive health care services are also essential to establish an optimized skill mix to deliver PHC and to facilitate nurses, midwives and others in working to their full scope of practice [24–26]. Accreditation systems can assist countries in responding to transformative education needs and establishing mechanisms to address quality and equity in education [27, 28]. The commitment to following competency-based curricula suggests the growing importance of knowledge, skills and behaviours necessary to provide comprehensive people-centred care, thereby improving health outcomes and the overall cost-effectiveness of health services.

The highest combined responses of “completed” and “in progress” were for the interventions, *Engaging professional associations in policy discussions* and *Strengthening collaborative practices at policy level*. Broad and collaborative engagement in policy dialogue, with leadership from professional associations, is central to decision-making and policy development. The *Nursing Now* campaign (2018-2020) is a facilitator of interdisciplinary engagements to launch national *Nursing Now* networks that focus on raising the profile of nursing to enhance country-level contributions to UHC [29]. An integrated policy approach to developing the nursing and midwifery workforce is central to strengthening PHC and achieving UHC. Decisions on investment can strengthen not only access and health outcomes (SDG 3), but also improve education (SDG 4), enable economic participation by women and youth in the workforce (SDG 5) and advance decent work and inclusive economic growth (SDG 8). The WHO has a mandate to facilitate the monitoring and mutual accountability of Member States to jointly agreed milestones and targets. This also extends to monitoring the uptake and implementation of associated normative guidance and policies. Such monitoring and accountability activities should be embedded within broader health workforce information systems and policy mechanisms and underpinned by interdisciplinary and intersectoral policy dialogue that is backed by robust and valid health workforce data.

Our study suggested relatively high levels of implementation (≥ 80% with “completed” and “in progress” combined) of the two interventions related to national health workforce data collection. While these interventions could include data from various heath information systems, the progressive implementation of NHWA by countries is ongoing and will be leveraged to produce the first-ever State of the World’s Nursing (SoWN) report. The SoWN report will provide quantitative technical descriptions of the national nursing and midwifery workforces along with qualitative policy analyses and a forward-facing agenda for the workforces.

Our findings contribute to the understanding of nurses and midwives as drivers of progress on key global health agendas, including UHC and integrated people-centred primary care services. Responses indicate levels of ≥ 80% on implementation (“in progress” and “completed” combined) of *Improving access to health care services* and *Ensuring integrated people-centred health services* [5, 9, 30]. While GCNMOs appear to focus on UHC and people-centred PHC, other development priorities were not explicitly mentioned. For example, while GCNMOs and other stakeholders committed to the “decent work” agenda, advocacy for investment in nursing and midwifery, collaboration and use of technology in the 2018 Triad Statement [31], progress on interventions relating to positive practice environments, national advocacy plans and web-based interprofessional collaboration were among the highest indicated as “not started”. These lagging areas represent opportunities for future policy development and intervention.

There were also mixed outcomes for the status of interventions related to policy and leadership development. One of the highest reported “not started” was, *Update or establish programmes for nursing and midwifery leadership programmes*. Programmes for leadership preparation and policy literacy or competency have been a long-standing challenge for nursing and midwifery [32, 33]. The WHO has outlined roles and responsibilities for GCNMOs [34] to ensure strong leadership at the national level; the International Council of Nurses’ Global Nurse Leadership Programme also contributes to developing the capacity of nurse leaders from around the world [35]. However, more locally relevant development programmes may be needed to broadly equip nurses and midwives at the grassroots with the right skills to lead policy- and decision-making platforms at all levels of health care governance [36, 37], particularly for women and youth. An example of this type of policy lever is highlighted again through the *Nursing Now* campaign’s Nightingale Challenge 2020 which urges employers of nurses to provide leadership and development training for young nurses and midwives in 2020 [33]. The aim is to have at least 20,000 young nurses and midwives benefitting from leadership programmes by 2020—the same year marking the 200th anniversary of the birth of Florence Nightingale and tagged the “International year of the nurse and the midwife” by the WHO [38].

Our findings support existing literature on nursing and midwifery workforce development and progress on advancing UHC and PHC. High levels of completion in interventions related to regulation, accreditation and engaging professional associations are aligned with the published outcomes of the African Regulatory Collaborative, a 5-year initiative (2011–2016) focused on advancing nursing regulation through intraprofessional collaboration [39–41]. The Nursing Education Partnership Initiative (NEPI) established in 2011 by the U.S. President’s Emergency Plan for AIDS Relief [42, 43] also focused on strengthening nursing education and competency-based curricula. A study using the WHO Guidelines on Transformative Education for Health Professionals [44] as a benchmark found relatively high rates of accreditation of nursing and midwifery education and training programmes [45]. Other studies also note that nursing leadership has an important role in advancing integrated people-centred PHC and substantially contributing to UHC and leaving no one behind [6, 20, 21].

This study has important limitations to note. While 35 GCNMOs or their representatives submitted their information, the findings may not be generalizable beyond the respondent countries. Secondly, the instrument was a self-reported questionnaire; thus, respondent bias may be present as the status of implementation of the interventions could not be independently verified by the researchers. Thirdly, we could not ascertain whether the status of the interventions reported as completed or in progress was attributable to policy guidance provided by the SDNM or whether it was triggered by other factors. The 3-point scale applied in this study is inherently limited in providing an understanding in detail of the level and nature of progress made in implementing the interventions. Lastly, because baseline measures are not available, the survey does not currently provide a sense of progress over time. Despite these limitations, it could serve as a baseline for future similar assessments. Two respondents noted that reporting on the presence or absence of an intervention is challenging in countries with a federated system of governance, due to variance in different jurisdictions or administrative units of policy and governance. Future research on the nursing and midwifery workforce would be improved by using data elements that are standardized, country-validated and can be pooled across countries for sub-regional, regional and global synthesis. Further research summarizing the existing evidence on nurses and midwives’ contributions to UHC and the SDGs should include evidence on their returns on investment as an advocacy tool to secure investments and drive progress across the SDGs.

## Conclusion

This is the first assessment conducted on the progress made by WHO Member States in the implementation of the SDNM. Overall, 35 GCNMOs or their representatives reported 74% of the 22 country-level interventions to be either “completed” or “in progress”. Our findings suggest that GCNMOs are taking leadership steps to advance the agendas for UHC and PHC through strengthened regulations, accreditation, engagement of professional associations and workforce data collection.

The results of this study echo those of earlier analyses to track progress towards the implementation of country-level commitments made in the context of similar global policy frameworks and mechanisms [46]. The process of identifying national commitments towards a global goal and policy framework can be instrumental in providing momentum contributing to domestic and international recognition and, sometimes, investments in workforce development [47]. Our study aligns with these concepts and aim to trigger similar interests and policy development benefits for the nursing and midwifery workforce.

Further, these findings provide an encouraging assessment that interventions are appropriately being targeted at the underlying determinants, at instituional and organizational level, of effective health workforce governance and leadership, as highlighted by exisitng literature [48]. Gaps in translating policy commitments into action however remain, and policy- and decision-makers (including GCNMOs) require greater support and effort to address lagging areas of nursing and midwifery development in their setting. A broad implication for nurses and midwives at practice levels is a renewed call to support country ownership and leadership by implementing these policy initiatives (particularly in lagging areas and as may pertain to local realities) and contribute to national accountability by tracking and reporting upstream on progress where such reporting systems are available. Countries’ reporting on the implementation of the SDNM and other HRH-related commitments will help reinforce the increasing use of evidence-based data for informed decision-making. This will help stakeholders in promoting intersectoral country-level policy dialogue and build strategic investments to enable nurses and midwives provide better health care and ultimately help achieve the health-related SDGs.

## Additional file

Additional file 1: Survey questionnaire. (PDF 169 kb)

## Data Availability

The dataset is available from the corresponding author on reasonable request.

## Abbreviations

ARC: African Health Profession Regulatory Collaborative for Nursing and Midwifery
GCNMO: Government Chief Nursing and Midwifery Officer
NEPI: Nurse Education Partnership Initiative
PHC: Primary health care
SDG: Sustainable Development Goal
SDNM: Global Strategic Directions for Strengthening Nursing and Midwifery 2016–2020
SoWN: State of the World’s Nursing
TA: Thematic area
UHC: Universal health coverage
WHA: World Health Assembly
WHO: World Health Organization
