# Correction to: Are the Global Strategic Directions for Strengthening Nursing and Midwifery 2016–2020 being implemented in countries? Findings from a cross-sectional analysis

**DOI:** 10.1186/s12960-020-00492-w

**Published:** 2020-07-17

**Authors:** Onyema Ajuebor, Carey McCarthy, Yin Li, Sumaya Mohamed Al-Blooshi, Nonhlanhla Makhanya, Giorgio Cometto

**Affiliations:** 1 Health Workforce Department, World Health Organization, 20 Avenue Appia, CH-1211 Geneva 27, Switzerland; 2 Nell Hodgson Woodruff School of Nursing, Emory University, Atlanta, USA; 3 Nursing Department, Ministry of Health and Prevention, Abu Dhabi, UAE; 4 National Department of Health, Pretoria, South Africa

**Correction to: Hum Resour Health (2019) 17:54**

**https://doi.org/10.1186/s12960-019-0392-2**

Following publication of the original article [1], the authors identified an error in Table 2. The correct table is given below.

**Table 2:** Percent of interventions reported as “not started”, “in progress” and “completed” by thematic area

Thematic Area	No. of interventions	No. of responders	Not started N ***(%)***	In progress N ***(%)***	Completed N ***(%)***	In progress & completed N ***(%)***
1. Ensuring an educated, competent and motivated nursing and midwifery workforce within effective and responsive health systems at all levels and settings	7	35	61 *(25%)*	123 *(50%)*	61 *(25%)*	184 *(75%)*
2. Optimizing policy development, effective leadership, management and governance	6	35	42 *(20%)*	122 *(58%)*	46 *(22%)*	168 *(80%)*
3. Working together to maximize the capacities and potentials of nurses and midwives through intra- and interprofessional collaborative partnerships, education and CPD	4	35	46 *(33%)*	77 *(59%)*	10 *(8%)*	87 *(67%)*
4. Mobilizing political will to invest in building effective evidence-based nursing and midwifery workforce development	5	35	53 *(30%)*	93 *(53%)*	30 *(17%)*	123 *(70%)*
**Overall from four thematic areas**	**22**	**35**	**200*(26%)***	**420*(55%)***	**150*(19%)***	**570*(74%)***
