# Supplementary material for: Are the Global Strategic Directions for Strengthening Nursing and Midwifery 2016–2020 being implemented in countries? Findings from a cross-sectional analysis
Source: Hum Resour Health. 2019 Jul 12;17:54. doi: 10.1186/s12960-019-0392-2 (PMC6626395; doi:10.1186/s12960-019-0392-2)
Supplement: Supplementary file 1 — Survey questionnaire. (PDF 169 kb) [file 12960_2019_392_MOESM1_ESM.pdf]

Progress review on the Global strategic directions for strengthening nursing and midwifery 2016–2020: Update on country perspectives

World Health Organization

The Global Strategic Directions for Strengthening Nursing and Midwifery 2016-2020 (SDNM) was launched at the 2016 GCNMO Forum in Geneva. The SDNM provides policy makers, practitioners and other stakeholders with a framework to enhance the capacity for nursing and midwifery development at national, regional and global levels.

This year, the GCNMO Forum will dedicate a session on national progress in the implementation of SDNM. The purpose of this instrument is to gather perspectives on the implementation of country level interventions laid out in the SDNM 2016-2017. Inputs from countries will support preparations for the Forum's related session as well as feed into the Secretariat's report to the 144th Executive Board (January 2019).

This exercise will need approximately 15 minutes to complete. Please let us know your views by Sunday 13 May 2018. Do not hesitate to contact us at workforce2030@who.int should you have any questions. To facilitate responding to your email, kindly include "SDNM progress review" in the subject line of your email.

Thank you in advance for your valuable inputs.

* 1. Which country do you represent?

* 2. What is your position and main responsibility?

3. What is your gender?

- Male
- Female

* 4. Have you used the SDNM (2016-2020) to guide nursing and midwifery development in your country/setting?

- Yes
- No

* 5. If Yes to question 4, how useful do you find the SDNM 2016-2020 for guiding nursing and midwifery development in your setting?

- [ ] Useful
- [ ] Uncertain
- [ ] Not useful

6. Please briefly indicate what aspects you find to be most useful and least useful.

* 7. For the following interventions in thematic area 1 (Ensuring an educated, competent and motivated nursing and midwifery workforce within effective and responsive health systems at all levels and in different settings). Please indicate level of progress in your setting

	Not started	In progress	Completed
Develop national costed plans for nursing and midwifery development with a minimum cycle of four to five years and an in-built monitoring and evaluation system	○	○	○
Integrate minimum data sets into national human resources for health observatories or HRH information systems as a source of evidence-based decisions for the nursing and midwifery workforce	○	○	○
Develop, adopt, support and monitor, quality management systems for nursing and midwifery services	○	○	○
Establish or strengthen and maintain national accreditation standards for nursing and midwifery education	○	○	○
Conduct a task analysis of the various cadres providing nursing and midwifery services to clarify their roles and scopes of practice	○	○	○

	Not started	In progress	Completed
Review and implement competency-based curricula for educators, student nurses and student midwives, and preclinical teachers, taking into account quantity, quality and relevance of the nursing and midwifery workforce to meet local and national changing health needs	○	○	○
Develop and implement a plan on improving working conditions to ensure positive practice environments	○	○	○

* 8. For the following interventions in thematic area 2 (Optimizing policy development, effective leadership, management and governance). Please indicate level of progress in your setting

	Not started	In progress	Completed
Advocate and set up mechanisms to raise the level of involvement of nurses and midwives in policy- and decision-making across the major sectors of service planning and management, education and management of human resources	○	○	○
Engage professional associations of nurses and midwives in policy discussions and development	○	○	○
Obtain resources, and where necessary, use regional support from WHO and competent national bodies, to update or establish programmes for leadership preparation in all sectors of nursing and midwifery responsibility	○	○	○

	Not started	In progress	Completed
Advocate effective systems of professional regulation, and strengthen and support the legislative authority to implement them	◯	◯	◯
Establish and maintain robust systems for assessing the appropriate implementation of nationally agreed nursing and midwifery practice standards in health care delivery systems	◯	◯	◯
Work to implement data collection and information systems to enable reliable reporting on the nursing and midwifery workforce status as relevant to local contexts, and to inform the national health workforce accounts	◯	◯	◯

* 9. For the following interventions in thematic area 3 (Working together to maximize the capacities and potentials of nurses and midwives through intra- and interprofessional collaborative partnerships, education and continuing professional development). Please indicate level of progress in your setting

	Not started	In progress	Completed
Formulate, strengthen and reinvigorate interdisciplinary and multisectoral technical working groups on interprofessional education and collaborative practice based on evidence	○	○	○
Strengthen collaborative practices at policy level to maximize effective nursing and midwifery input on health care	○	○	○
Develop or strengthen national nursing and midwifery strategies on interprofessional education and collaborative practice	○	○	○
Create interprofessional networks facilitated through web-based communities of practice to improve the quality of education, safety of practice and capacities of the nursing and midwifery workforce	○	○	○

* 10. For the following interventions in thematic area 4 (Mobilizing political will to invest in building effective evidence-based nursing and midwifery workforce development). Please indicate level of progress in your setting

	Not started	In progress	Completed
Formulate and implement nursing and midwifery policies that ensure integrated people-centred services that are in line with universal health coverage and the Sustainable Development Goals	◯	◯	◯
Establish a multisectoral group to support the development of nursing and midwifery policies	◯	◯	◯
Develop and support nursing and midwifery interventions that lead to improved access to health care services through the creation of links among the public, nongovernmental and private sectors to minimize barriers obstructing access to health services for vulnerable populations in urban, rural and remote areas	◯	◯	◯
Update nursing and midwifery curricula and ensure that nursing and midwifery students acquire effective leadership skills, including assertiveness, negotiation and advocacy, and ability to develop and influence health policy	◯	◯	◯
Develop and implement national advocacy plans targeting policy-makers and organizations	◯	◯	◯

11. If needed, please provide any additional comments on the interventions above

* 12. Regional framework for nursing and midwifery development

- [ ] We have one and we implement it
- [ ] We have one but do NOT implement it
- [ ] We do not have one

* 13. Outline three priority needs for nursing and midwifery development in your setting?

* 14. Outline three priority areas of support for nursing and midwifery development in your setting?

15. List major successes achieved in the last two years (2016-2018)

16. Please provide two case studies of successful implementation in your country/setting. Please provide citation if available.
